# Long-Term Impact of Western Diet on Right Ventricular Transcriptome: Uncovering Sex-Specific Patterns in C57BL/6J Mice

**DOI:** 10.3390/ijms27010259

**Published:** 2025-12-26

**Authors:** Ani Stepanyan, Siras Hakobyan, Agnieszka Brojakowska, Malik Bisserier, Roksana Zakharyan, Suren Davitavyan, Tamara Sirunyan, Gisane Khachatryan, Mary K. Khlgatian, Shihong Zhang, Ania Baghoomian, Susmita Sahoo, Lahouaria Hadri, Venkata Naga Srikanth Garikipati, Arsen Arakelyan, David A. Goukassian

**Affiliations:** 1 Institute of Molecular Biology, National Academy of Science of the Republic of Armenia, Yerevan 0014, Armenia; 2 Cardiovascular Research Institute, Icahn School of Medicine at Mount Sinai, New York, NY 10029, USA; 3 Department of Cell Biology, Yale School of Medicine, New Haven, CT 06510, USA; 4 Department of Cell Biology and Anatomy and Physiology, New York Medical College, Valhalla, NY 10595, USA; 5 Department of Pharmacological Sciences, Icahn School of Medicine at Mount Sinai, New York, NY 10029, USA; 6 Aging + Cardiovascular Discovery Center, Department of Cardiovascular Sciences Lewis Katz School of Medicine, Temple University, Philadelphia, PA 19140, USA

**Keywords:** Western diet, right ventricle, transcriptome, sex

## Abstract

The Western diet (WD) has been linked to various structural and functional alterations in the left ventricle (LV), but the molecular response of the right ventricle (RV) remains largely unknown. Given the RV’s distinct anatomical and functional characteristics, it is crucial to understand how long-term WD exposure affects RV gene expression, especially in a sex-specific context. Our objective was to perform gene expression profiling of the RV late responses to WD in wild-type mice. Male and female C57BL/6J mice were fed a WD for 125 days from 300 to 425 days of age, and RV tissues were collected at 530 and 640/750 (female/male) days. mRNA sequencing was performed on RV tissues to identify differentially expressed genes (DEGs) between WD-fed and normal diet (ND)-fed groups. Data processing and analysis were conducted using the STAR aligner and DESeq2. WD-induced RV transcriptomic changes were characterized by differential expression of genes associated with cardiac remodeling and transcriptional regulation in both sexes. In females, additional genes showing altered expression were associated with immune response, whereas in males, changes were more limited, primarily involving genes related to circadian rhythm and cardiac remodeling. Echocardiography revealed modest, sex-specific differences: WD-fed females showed a decrease in right-ventricular internal diameter in diastole and a trend toward increased pulmonary trunk diameter, whereas males showed no notable changes. These exploratory results suggest that WD is associated with modest transcriptomic changes in the RV in both sexes, with only minor structural differences observed in females, indicating subtle sex-specific effects after a switch to normal chow.

## 1. Introduction

The Western diet (WD) has been widely associated with an increased risk of cardiovascular diseases (CVD) and is known to contribute to cardiac structural alterations and impaired function. While numerous studies have demonstrated the WD-associated functional, structural, and transcriptomic alterations in the left ventricle (LV) [1,2,3], the specific impact of WD on right ventricular (RV) structure and function, particularly in the context of gene expression in RV tissue, remains poorly understood. A significant knowledge gap remains on whether WD induces intrinsic, RV-specific molecular remodeling or whether the observed changes are secondary to systemic metabolic disturbances and pulmonary vascular remodeling. Clarifying this distinction is essential for understanding the direct impact of dietary factors on RV pathophysiology and for developing targeted therapeutic strategies. These two cardiac chambers are distinctly characterized by their different developmental origins, structural makeup, cellular profiles, and, as a result, their functional responses to varying hemodynamic environments [4]. Given these biological differences, it is important to investigate whether the RV exhibits unique vulnerabilities to diet-induced metabolic stress. Moreover, it has been demonstrated that RV functional impairment is a key predictor of heart failure, pulmonary hypertension, mortality, and poor outcomes in LV-related diseases [5]. This highlights the need to investigate the molecular signatures underlying conditions associated with RV dysfunction and emphasizes the clinical relevance of understanding RV-specific pathophysiology, particularly in highly prevalent conditions such as those induced by WD. Few animal studies have established a link between WD, metabolic stress, and hemodynamic stress in the RV [6]. A high-fat WD was shown to differentially affect lipid deposition in the LV and RV, resulting in more pronounced effects on RV diastolic function compared to systolic function in male C57Bl/6 mice [6]. In a recent study, total RNA sequencing of the RV revealed upregulation of MAPK8, increased expression of the extracellular matrix (ECM) component fibronectin, and abnormal cytokine production, along with T-cell proliferation, in a WD-fed preclinical model of cardiometabolic heart failure in female Ossabaw swine [7]. Despite these findings, the comprehensive characterization of RV-specific transcriptional adaptations to WD remains limited. Moreover, the long-term or persistent effects of diet withdrawal on these adaptations remain poorly understood. There is a notable gap in research regarding sex-based differences in RV responses to WD despite well-documented differences in cardiac metabolism, immune signaling, and stress response pathways between males and females [8,9,10]. Therefore, a thorough investigation of WD-induced changes in the RV function of both sexes, including long-term effects, is crucial for understanding the full extent of risks that high-fat diets pose on RV function. Long-term studies are particularly important, as WD can induce lasting metabolic and epigenetic changes that may persist even after a return to healthier dietary conditions [11,12].

We previously reported distinct sex-specific and age-associated differences in the LV transcriptome signature between WD-fed male and female C57BL/6J mice [3]. These findings suggest that sex may influence cardiac transcriptional responses to metabolic stress, further supporting the importance of investigating sex-specific differences in the RV. The current study involved the same animals that were fed a WD for a relatively long duration (125 days), with potential alterations examined in the later stages of their lives, specifically at 125 and 215/325 (female/male) days following the termination of WD and switch to normal mouse chow. Our goal was to perform gene expression profiling of the RV late responses to WD in male and female wild-type C57BL/6J mice and to identify genes or processes with the greatest sex-linked changes in RV tissue. By employing this experimental framework, our study directly addresses these critical gaps by delineating RV-specific, sex-dependent, and persistent transcriptional consequences of WD exposure.

## 2. Results

**Figure 1:**
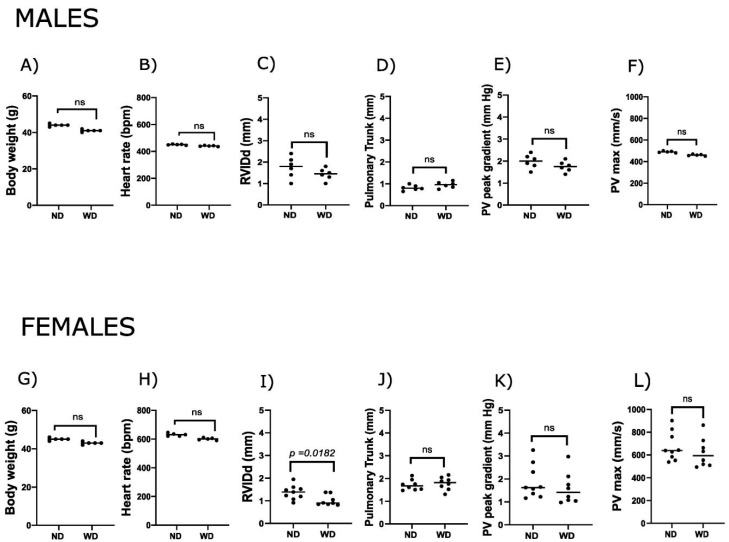
Right ventricular response to a Western diet in male and female mice. Panels show physiological and echocardiographic (Doppler) parameters in C57BL/6J male (750 days old) and female (640 days old) mice fed with a normal diet (ND) and a Western diet (WD). Male data are shown in panels (**A**–**F**): (**A**) body weight, (**B**) heart rate, (**C**) right ventricular internal diameter in diastole (RVIDd), (**D**) pulmonary trunk diameter, (**E**) pulmonary valve peak gradient (Doppler), and (**F**) pulmonary valve maximal velocity (PVmax, Doppler). Female data are shown in panels (**G**–**L**): (**G**) body weight, (**H**) heart rate, (**I**) RVIDd, (**J**) pulmonary trunk diameter, (**K**) pulmonary valve peak gradient, and (**L**) PVmax. N = 7–10 animals per group; *p*-values were calculated using an unpaired *t*-test, and *p* < 0.05 was considered statistically significant.

Echocardiographic analysis revealed no significant differences in RV structure or function between the normal diet (ND) and WD groups in male mice, with all measured parameters, including body weight, heart rate, pulmonary trunk diameter, pulmonary valve peak gradient, and pulmonary valve peak velocity (PVmax), being similar between the groups. In female mice, right ventricular internal diameter in diastole (RVIDd) was significantly decreased in WD-fed animals compared to ND controls (*p* < 0.02) (Figure 1C,I). While not statistically significant, we observed slightly larger pulmonary trunk diameter and lower PVmax and peak gradient in both sexes (Figure 1). Overall, the observations in females may reflect minor diet-related alterations in RV measurements, whereas no clear effects were observed in males.

**Figure 2:**
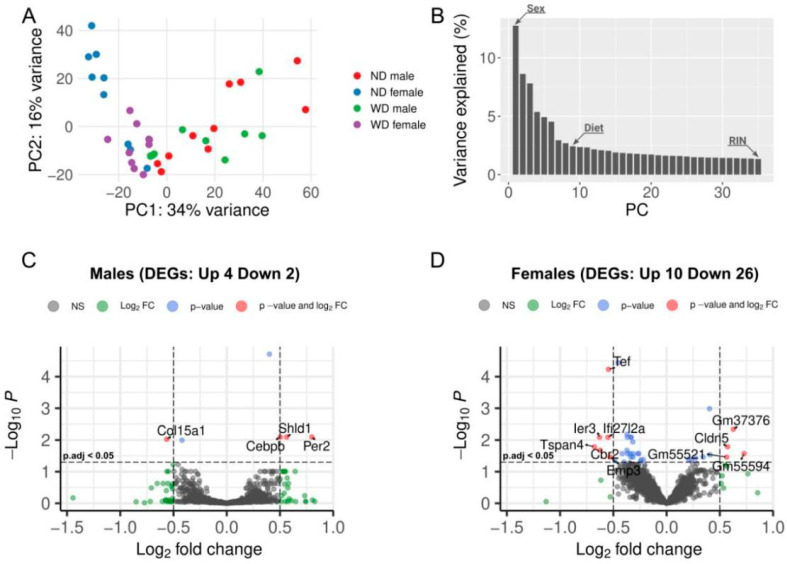
Global and sex-specific gene expression patterns: principal component analysis (PCA) and volcano plots for males and females: (**A**) Each dot in the PCA plot represents a sample, with colors indicating sex and dietary pattern (normal diet (ND) female/Western diet (WD) female: blue/purple; ND male/WD male: red/green). (**B**) Elbow plot showing the percentage of variance explained by each principal component (PC). Arrow marks on the plot bars indicate the clinical groups significantly associated with the corresponding principal components. (**C**,**D**). The volcano plots show the number of differentially expressed genes (DEGs) in males (**C**) and females (**D**) before excluding RIN-associated DEGs.

Principal component analysis (PCA) was performed to assess the global variability in our samples. The separation of samples into clusters representing each sex indicated different transcriptome profiles in the RV of male and female mice (Figure 2A). ANOVA test on principal components indicated that sex and diet corresponded to the first and eighth principal components (PC), accounting for 34.2% and 1.2% of the variability in the expression data, respectively, while RIN was associated with the thirty-fifth PC, explaining 0.4% of the data variability (Figure 2B). However, the RIN values were substantially different between some experimental groups (Supplementary Figure S1); therefore, we combined samples from different collection time points and performed differential expression analysis between normal and WD-fed groups, irrespective of time. Further, to evaluate whether sample collection age contributed to transcriptomic variation, we performed exploratory PCA and heatmap analyses stratified by sex, diet, and age (530 days; 640/750 days). These analyses suggested some age-related effects; however, age alone did not explain the separation observed between the WD and ND groups (Supplementary Figures S2 and S3).

Differential gene expression analysis between the WD and ND groups identified six differentially expressed genes (DEGs) in male animals, of which four (*Rhobtb1*, *Shld1*, *Per2*, and *Cebpb*) were upregulated, and two (*Col15a1*, *Cd93*) were downregulated. One of the upregulated genes (*Shld1*) in males overlapped with RIN-significant DEGs and was removed from the final list (Table 1, Figure 2C).

In female animals, 36 DEGs were detected, with 10 genes upregulated and 26 genes downregulated (Figure 2D). After filtering out RIN-significant genes, six upregulated and 15 downregulated DEGs remained (Table 1).

No overlapping DEGs were identified between males and females, indicating distinct biological responses to WD exposure between the sexes.

In our previous study, we identified significant transcriptomic changes in the LV heart tissue of WD-fed animals [3]. We examined overlapping DEGs between the LV and RV tissues to identify shared molecular alterations between heart chambers. The *Col15a1* gene was downregulated in both LV and RV chambers of WD-fed male animals. In females, two commonly downregulated genes were also identified: *Tspan4* and *Angptl2*.

Overrepresentation analysis of DEGs in females revealed no significant pathway enrichment, suggesting that the observed transcriptomic changes may be more dispersed across different biological processes rather than concentrated within specific pathways.

## 3. Discussion

To our knowledge, this study is the first to explore the long-term effects of WD on RV tissue. We found that WD-fed mice exhibited modest and limited changes in RV gene expression, which may be consistent with subtle transcriptional patterns sometimes associated with cardiac remodeling, although our results do not indicate extensive remodeling. In females, these transcriptomic alterations involved genes linked to cardiac stress, inflammation, and energy metabolism, whereas males showed more limited changes, primarily in genes associated with remodeling and circadian rhythm. Echocardiography assessments supported a reduction in RVIDd without changes in pulmonary trunk diameter, PVmax, or PV peak gradient, while males showed no significant differences across parameters. These observations may indicate sex-biased tendencies in RV response, though the magnitude of these differences remains modest and driven by a limited number of DEGs and a single significant echocardiographic parameter. Future studies incorporating right-heart catheterization and Doppler echocardiography will be essential to directly assess hemodynamics, RV function, and pulmonary vascular flow, and to determine whether WD contributes to pulmonary hypertension or RV outflow tract obstruction.

**Figure 3:**
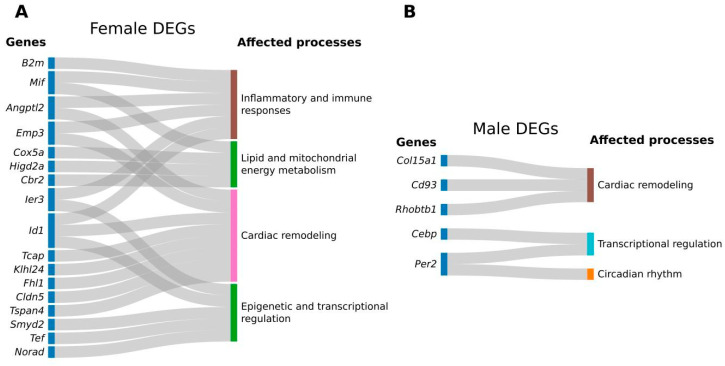
Differentially regulated genes and corresponding processes in comparison groups. Two Sankey plots represent the following comparisons: normal diet (ND) vs. Western diet (WD) male mice (**A**), ND vs. WD female mice (**B**).

We identified several dysregulated genes in female RV related to the ECM, either through structural roles (*Tcap, Klhl24, Fhl1,* and *Cldn5*), remodeling (*Angptl2, Id1*), and cell adhesion (*Tspan4, Emp3*) (Figure 3A). A recent study reported that fibronectin is dysregulated in the RV of WD-fed female Ossabaw swine in a preclinical model of cardiometabolic HF after 10 months of WD feeding, suggesting the role of altered ECM regulation in the RV alterations induced by WD [7]. While previous work has identified dysregulation of ECM-related genes during WD feeding, our study reveals that after transitioning to an ND, a distinct set of ECM-associated genes remains dysregulated. These findings raise the possibility that early dietary stress may have lingering molecular effects in the RV, although the modest scale of these transcriptomic shifts warrants further verification. Although the genes identified in our study in female RV are not direct ECM components, they significantly influence ECM remodeling, fibrosis, and cellular interactions with the matrix [13,14]. Importantly, most of these dysregulated genes are known to play a role in HF [15,16,17,18,19]. These changes may reflect compensatory changes in female RV tissue in response to functional alterations caused by early-life WD feeding [20]. Alternatively, they could represent early molecular signatures of cardiac dysfunction or persistent epigenetic and transcriptional reprogramming following dietary transition. Given the small number of DEGs, the absence of significant pathway enrichment, and minimal echocardiographic differences, our results should be interpreted as modest transcriptional changes rather than indicative of broad remodeling. Further studies are needed to validate these findings and elucidate the underlying mechanisms.

In male mice, we observed that the ECM component Collagen Type XV Alpha 1 Chain encoding gene (*Col15a1*) was downregulated in the RV in response to WD (Figure 3B). Col15a1 is a non-fibrillar collagen that plays a crucial role in the ECM of cardiac tissue, especially during cardiac remodeling [21,22]. Recent research has highlighted its involvement in various aspects of cardiac remodeling, including fibrosis and tissue repair [23]. Additionally, we found two genes known for their potential roles in cardiac and vascular remodeling, *Rhobtb1* and *Cd93*, to be dysregulated in the male RV [24,25]. These findings suggest that both female and male right ventricles exhibit limited transcriptional shifts in remodeling-related genes, though the small number of DEGs prevents strong conclusions. However, males showed less pronounced transcriptional changes, potentially reflecting biological differences in compensatory mechanisms or limited statistical power. Corresponding echocardiographic assessments revealed subtle, sex-specific differences: WD-fed females exhibited a significant decrease in RVIDd, whereas males showed no significant change. In both sexes, there were trends toward increased pulmonary trunk diameter and modest reductions in peak gradient. These findings align with previous reports that WD can influence RV structure and diastolic function in mice [6].

Our results indicate that female mice exhibited long-term transcriptomic alterations in the RV, affecting genes associated with inflammatory and immune responses (*Ier3, Angptl2, Mif, B2m, Id1,* and *Emp3*), as well as lipid and mitochondrial energy metabolism (*Cox5a, Higd2a,* and *Cbr2*) [26,27,28,29,30,31]. A longitudinal study showed that a Westernized diet induces inflammation in the heart and white adipose tissue, suggesting potential contributions of immune cells to the observed low-grade inflammatory response [32]. The growing evidence shows that obesity and inflammation are linked to an increased risk of CVD [33,34]. Anti-inflammatory treatments targeting various signaling pathways and regulators are used to address maladaptive RV responses [35]. This study expands the knowledge of immune targets for treatment strategies in RV failure due to prolonged WD diets, which may affect lipid metabolism. Although several DEGs were annotated to immune or metabolic pathways, the lack of pathway-level enrichment suggests that these changes should be interpreted as exploratory.

Our findings align with previous research on sex-specific transcriptomic signatures of decompensated RVs, particularly regarding the dysregulation of immune response and lipid metabolism in females [36]. Prior studies shown that male decompensated RVs exhibit upregulation of pathways related to calcium signaling, amino acid metabolism, and histone demethylation, whereas females demonstrated enrichment of genes involved in inflammatory responses and fatty acid β-oxidation [36]. In this context, the sex-associated differences observed in our study should be interpreted as biologically plausible rather than direct mechanistic assertions. These differences may reflect, but cannot be attributed solely to known variations in metabolic, hormonal, and epigenetic factors between males and females [32,33]. This interpretation is supported by growing evidence that males and females exhibit distinct lipid metabolism, mitochondrial function, metabolic responses to diet, and epigenetic regulation of metabolism and immune responses [37,38,39,40].

Compared to our previous findings on the LV long-term response to WD, the RV shows relatively weaker transcriptional alterations, and the DEGs found in the RV of WD-fed male and female mice are distinct from those found in the LV. Moreover, compared with the previously reported LV response, the current study identified several altered genes related to the immune response in the female RV of WD-fed mice [3]. WD has been shown to impair glucose tolerance and cardiac lipid dynamics, preceding HF with reduced ejection fraction (HFrEF) in mice [1]. WD-induced dysregulation of fatty acid metabolism also disrupts metabolic remodeling and contractile function in the LV, contributing to cardiac hypertrophy [41]. High-fat diet-induced obesity alters the expression of 184 LV genes, highlighting dysregulation of glucose metabolism via Nr4a1 [42]. Additionally, diets high in fat and sucrose increase cardiac fibrosis and alter gene expression in mice and primates [43]. These studies underscore the systemic impact of WD on cardiac gene expression and situate our RV findings within the broader landscape of WD-induced cardiac remodeling.

Several previous transcriptomic studies have reported RV-specific gene networks in both healthy and failing hearts [4,36]. In particular, cardiac mesenchymal stromal cells from the RV and LV exhibit distinct gene expression profiles related to cardiac remodeling, energy metabolism, inflammation and cytokine responses, electrical conduction, and tissue repair [4,36]. These differences in the long-term transcriptomic response to WD might reflect structural and, consequently, functional differences between the two cardiac chambers [4]. For clarity, these statements provide biological context only and do not represent any direct LV-RV transcriptomic comparison in our data. The RV exhibits unique transcriptional patterns, operates under low pressure and low resistance, and distinctly adapts to WD-induced metabolic stress [6,44].

The molecular patterns observed in our study parallel clinical evidence that metabolic and inflammatory states associated with obesity and the metabolic syndrome are important modulators of RV structure and function in humans. Population-based studies such as the MESA-RV cohort demonstrated that increased adiposity and metabolic perturbations correlate with increased RV mass, larger volumes, and altered RV function [45]. In contrast, short-term dietary manipulations have been shown to acutely affect RV diastolic function in healthy subjects [46]. Furthermore, adipokines and pericardial fat, which are mediators of systemic metabolic inflammation, are associated with adverse RV remodeling in community cohorts [47]. In females, several altered genes are linked to immune and metabolic pathways, consistent with evidence that systemic inflammation, insulin resistance, and dyslipidemia in metabolic syndrome promote pulmonary vascular remodeling and increase afterload on the RV, thereby driving maladaptive remodeling [48]. In males, we observed dysregulation of genes associated with circadian regulation, which is clinically relevant given that circadian disruption has been implicated in adverse cardiac remodeling and hypertrophy in human studies of failing ventricles [49]. Taken together, the gene-level changes identified in our study map onto pathways central to human RV vulnerability and dysfunction, reinforcing the translational relevance of our findings. Comparison of DEGs identified in our study with remodeled human RV transcriptomes revealed that most of these genes were not detected, suggesting that while the implicated pathways (ECM, endothelial signaling, inflammation, metabolism, and transcription) are biologically plausible, the specific gene changes may be species- or context-specific. This emphasizes the need for caution when claiming direct translational overlap and highlights the importance of further targeted validation in human RV tissue or circulating biomarkers. More broadly, these parallels indicate that WD-induced molecular signatures in mice align with systemic metabolic and inflammatory mechanisms known to contribute to RV vulnerability in humans. This suggests that diet-related stressors may prime the human RV for maladaptive remodeling and warrant future studies integrating dietary patterns with RV imaging and biomarker profiling.

Our results support the hypothesis that early-life nutritional habits can induce lasting molecular changes in the heart even long after switching to a normal diet. These findings have potential implications for both basic science and clinical practice. The persistent effects of a WD highlight the need for early dietary intervention, personalized treatment strategies, and further investigation of epigenetic mechanisms and recovery pathways. The observed sex-specific variations highlight the importance of tailored approaches to understanding and managing RV dysfunction under dietary stress conditions. Lastly, from a public health perspective, these findings reinforce the value of promoting dietary patterns aligned with ancestral eating habits to reduce CVD risk and healthcare costs.

We acknowledge several limitations in our study. The primary limitation is the variability in RIN across experimental groups. To improve statistical power, we combined age groups within treatment conditions and by sex. Furthermore, we were unable to compare the LV and RV transcriptome signatures between experimental groups, because, although tissues derived from the same animals, the libraries were sequenced in separate runs. Another limitation is the use of a chow diet as the control, which introduces potential batch-to-batch variability due to its natural ingredient composition. Our findings are further limited by the absence of protein-level or histological validation, which constrains the interpretation of transcriptomic changes in terms of structural and functional remodeling. Future studies with larger sample sizes are needed to investigate DNA methylation and protein expression changes, providing a more comprehensive understanding of the mechanisms underlying the RV response to WD. Finally, the relatively small number of DEGs observed in males should be interpreted with caution, as this limits the statistical power and reliability of sex-specific comparisons.

## 4. Materials and Methods

### 4.1. Animal and Diet

All animal procedures were performed following the standards of the Guide for the Care and Use of Laboratory Animals for the National Institutes of Health and approved by the Animal Care and Use Committees at Brookhaven National Laboratory (BNL) (Upton, NY) (BNL IACUC Protocol #502) and the Icahn School of Medicine at Mount Sinai (NY, NY) (ISMMS IACUC Protocol #2019-0017). All animal experiments were conducted at the Brookhaven National Laboratory Animal Facility (Upton, NY, USA). The animal room was kept at a 12:12 h light-dark cycle at 20–22 °C with 30–70% relative humidity. Animals were monitored at least once a day for any physical or behavioral changes that might indicate distress, discomfort, pain, or injury. Only mice showing general signs of poor health and distress (e.g., rapid heart/respiratory rate, oral/nasal discharge, wound dehiscence, marked swelling, neoplasms >2 cm or ulcerating, inability to eat/drink, or weight loss > 15%) were immediately euthanized using 100% CO_2_ at 20% air replacement per minute rate, followed by cervical dislocation.

The mice used in this study are the same ones we previously studied to investigate the long-term effects of a Western diet on LV tissue [3]. The study groups consist of C57BL6/J mice, which are more resistant than genetically modified murine atherosclerosis models, Apolipoprotein E (ApoE), or low-density lipoprotein receptor (LDLR) knockout mice, to develop atherosclerosis. Mice were 90 days old when purchased from Jackson Labs. Normal diet (ND)-fed (n = 20, male/female:10/10) mice were fed a standard chow diet ad libitum (LabDiet^®^ 5015, Supplementary Table S1). Chow diets, which are grain-based and composed of natural ingredients such as soybean meal and fish meal, are inherently subject to compositional variability across batches. This batch-to-batch variation can lead to fluctuations in the levels of nutrients, including phytoestrogens and other bioactive compounds, which may influence metabolic and physiological responses in rodents [50,51,52]. Despite these limitations, we selected a chow diet as the control for several reasons. Primarily, chow diets are extensively utilized in rodent research, providing a broad base of comparative data. Furthermore, their complex and diverse composition may more accurately reflect the heterogeneous nutrient exposure encountered in natural settings, thereby offering greater physiological relevance. Although batch variability is a recognized constraint, the two-year longitudinal design of our study necessitated prioritization of dietary freshness—an important factor that has been shown to significantly affect nutrient composition. The WD-fed group (n = 20, male/female:10/10) was fed a custom Teklad diet containing 42% fat (TD.88137, Supplementary Table S2) (Envigo, Madison, WI) for 125 days from ages 300 to 425 days. RV tissues were collected at 530 and 640/750 (female/male) days of age [3]. Details on animal exclusions, mortality, and autopsy findings for both sexes were previously reported [3].

### 4.2. Physiological Measurements and Echocardiography

C57BL/6J male (750 days old) and female (640 days old) mice body weight (g) was measured using a digital scale. Heart rate (beats per minute) was recorded during echocardiography. Transthoracic echocardiography with Doppler was performed to assess RV structure and pulmonary hemodynamics. Mice were anesthetized with isoflurane (induced at 3%, maintained at 1–2%), hair was removed from the neckline to the mid-chest, and mice were placed supine on a heated platform to maintain a core temperature of 37 °C. Two-dimensional and M-mode images were obtained to measure right ventricular internal diameter in diastole (RVIDd, mm) and pulmonary trunk diameter (mm). Doppler echocardiography at the pulmonary valve was used to measure pulmonary valve peak gradient (mm Hg) and pulmonary valve maximal velocity (PVmax, mm/s). Each echocardiographic parameter is reported as the average of three measurements.

### 4.3. mRNA Library Preparation and Sequencing

NEBNextUltra™II RNA Library Prep Kit (New England Biolabs (NEB), Ipswich, MA, USA) was used for the Poly-A selected mRNA library preparation. The RNA integrity number (RIN) of the RNA samples, concentration, and the quality check of libraries were performed using an Agilent Bioanalyzer (Agilent Technologies, Santa Clara, CA, USA) and qPCR. The sequencing was performed on the Illumina NovaSeq-6000 (Illumina Inc., San Diego, CA, USA) platform with an average (mean ± SD) of 5.0 × 10^7^ ± 6.8 × 10^6^ paired-end reads per sample. Detailed RNA sequencing QC data are presented in Supplementary Table S3.

### 4.4. Data Processing and Statistics

FastQC was used to assess the quality of raw sequencing reads. The reads were then aligned to the mouse reference genome (mm39) using the STAR aligner (version 2.7.8a). Because low RNA integrity numbers (RIN) were observed for some samples, we implemented a custom gene counting method suggested by Sigurgeirsson et al., which improves the accuracy of gene expression quantification in degraded RNA samples [53]. We generated a custom GTF annotation file that retained only the 500 bp region from the 3′ end of each gene. Gene read counts were then calculated using HTSeq-count (version 1.99.2) with this custom GTF file [54].

Raw read counts were filtered to retain only genes with more than 20 counts in all samples. We performed a principal component analysis (PCA) on the normalized gene expression data and used eigenvalues to conduct an ANOVA test to identify principal components (PCs) that are significantly associated with sample annotations. Differential gene expression analysis was performed using the DESeq2 R package (version 1.42.0) on filtered read counts [55]. Since some experimental groups had low RIN values (Supplementary Figure S1), we combined samples from different collection time points and performed differential expression analysis between normal and WD-fed groups, irrespective of time. Genes with an FDR < 0.05 were considered differentially expressed. Further, to minimize false positives caused by library quality issues, we excluded genes whose differential expression was primarily driven by RIN values. Finally, an overrepresentation analysis of DEGs was conducted using the Enrichr R package (version 3.2) [56]. FDR adjusted *p* values < 0.05 were considered significant.

## 5. Conclusions

WD induced modest yet persistent transcriptomic alterations in the RV of C57BL/6J mice, even after the diet was discontinued. These changes primarily involved genes related to cardiac remodeling, with females showing alterations in immune response and mitochondrial energy metabolism, while males exhibited fewer changes. Overall, these findings suggest that WD elicits limited but detectable sex-biased transcriptomic responses in the RV.

## Figures and Tables

**Table 1:** Differentially expressed genes in the RV tissue of WD-fed male and female mice.

Gene Type	Gene Name	Gene Description	Log2FC	padj
**MALES**				
protein_coding	*Per2*	period circadian clock 2	0.799	8.02 × 10^−3^
protein_coding	*Cebpb*	CCAAT/enhancer binding protein (C/EBP), beta	0.502	8.02 × 10^−3^
protein_coding	*Rhobtb1*	Rho-related BTB domain-containing 1	0.401	1.94 × 10^−5^
protein_coding	*Col15a1*	collagen, type XV, alpha 1	−0.564	9.42 × 10^−2^
protein_coding	*Cd93*	CD93 antigen	−0.420	1.01 × 10^−2^
**FEMALES**				
lncRNA	*Gm55594*	predicted gene, 55594	0.728	2.69 × 10^−2^
protein_coding	*Cldn5*	claudin 5	0.573	1.63 × 10^−2^
protein_coding	*Fhl1*	Four and a half LIM domains 1	0.408	2.94 × 10^−2^
protein_coding	*Mtus2*	microtubule-associated tumor suppressor candidate 2	0.344	3.46 × 10^−2^
protein_coding	*Klhl24*	Kelch-like 24	0.273	3.75 × 10^−2^
lncRNA	*Norad*	non-coding RNA activated by DNA damage	0.234	4.23 × 10^−2^
protein_coding	*Tspan4*	tetraspanin 4	−0.678	1.64 × 10^−2^
protein_coding	*Ier3*	immediate early response 3	−0.630	8.13 × 10^−3^
protein_coding	*Cbr2*	carbonyl reductase 2	−0.624	2.11 × 10^−2^
protein_coding	*Tef*	thyrotropin embryonic factor	−0.546	5.87 × 10^−5^
protein_coding	*Emp3*	epithelial membrane protein 3	−0.532	3.46 × 10^−2^
protein_coding	*Tcap*	titin-cap	−0.454	3.65 × 10^−5^
protein_coding	*Id1*	inhibitor of DNA binding 1, HLH protein	−0.372	2.11 × 10^−2^
protein_coding	*Angptl2*	angiopoietin-like 2	−0.368	8.13 × 10^−3^
protein_coding	*Cox5a*	cytochrome c oxidase subunit 5A	−0.368	2.69 × 10^−2^
protein_coding	*B2m*	beta-2 microglobulin	−0.342	2.69 × 10^−2^
protein_coding	*Mif*	macrophage migration inhibitory factor	−0.335	8.13 × 10^−3^
protein_coding	*Higd2a*	HIG1 domain family, member 2A	−0.316	3.46 × 10^−2^
protein_coding	*Hypk*	Huntingtin-interacting protein K	−0.261	2.70 × 10^−2^
protein_coding	*Cuta*	cutA divalent cation tolerance homolog	−0.246	4.93 × 10^−2^
protein_coding	*Smyd2*	SET and MYND domain-containing 2	−0.239	4.23 × 10^−2^

## Data Availability

The sequencing data are available in the Gene Expression Omnibus (GEO) under accession number GSE300189.

## Supplementary Materials

The following supporting information can be downloaded at: https://www.mdpi.com/article/10.3390/ijms27010259/s1.
