# Supplementary material for: Long-Term Impact of Western Diet on Right Ventricular Transcriptome: Uncovering Sex-Specific Patterns in C57BL/6J Mice
Source: Int J Mol Sci. 2025 Dec 26;27(1):259. doi: 10.3390/ijms27010259 (PMC12785519; doi:10.3390/ijms27010259)
Supplement: Supplementary file 1 [file ijms-27-00259-s001.zip › ijms-4035266-supplementary.pdf]

"Long-term Impact of Western Diet on Right Ventricular Transcriptome: Uncovering Sex-Specific Patterns in C57BL/6J Mice" – Stepanyan et al.

The supplementary file includes **Table S1** and **Table S2**, detailing the composition of the chow and Western diets used in the experimental groups, respectively. **Table S3** provides comprehensive information on RNA sample quality control, sequencing data metrics, and the age, sex, and treatment details for each sample. Additionally, **Figure S1** shows the distribution of RNA integrity numbers (RIN) across all groups, and **Figure S2** present the Principal Component Analysis stratified by diet, sex, and collection age. **Figure S3** visualizes the top 50 highly variable genes in a heatmap, grouped by diet, sex, and collection age.

Table S1. Composition of the Standard Chow Diet (LabDiet® 5015) used in experimental groups.

Category	Nutrient/Component[1]	Amount
**Macronutrients and General Content[2]**	Crude Protein	19.0 %
	Fat (ether extract)	11.1 %
	Fat (acid hydrolysis)	12.0 %
	Crude Fiber	2.3 %
	Neutral Detergent Fiber (NDF)[3]	10.1 %
	Acid Detergent Fiber (ADF)[4]	2.9 %
	Nitrogen-Free Extract	51.7 %
	Starch	33.5 %
	Sucrose	0.90 %
	Total Digestible Nutrients	85.2 %
	Gross Energy	4.74 kcal/g
	Physiological Fuel Value[5]	3.83 kcal/g
	Metabolizable Energy	3.58 kcal/g
**Amino Acids**	Arginine	1.16 %
	Cystine	0.37 %
	Glycine	0.81 %
	Histidine	0.47 %
	Isoleucine	0.85 %
	Leucine	1.43 %
	Lysine	1.05 %
	Methionine	0.61 %
	Phenylalanine	0.87 %
	Tyrosine	0.55 %
	Threonine	0.72 %
	Tryptophan	0.24 %
	Valine	0.90 %
	Serine	1.01 %
	Aspartic Acid	2.04 %
	Glutamic Acid	4.18 %
	Alanine	1.00 %
	Proline	1.30 %
	Taurine	0.00 %
**Fatty Acids & Lipids**	Linoleic Acid	2.08 %
	Linolenic Acid	0.16 %
	Arachidonic Acid	0.03 %
	Omega-3 Fatty Acids	0.21 %
	Total Saturated Fatty Acids	3.70 %
	Total Monounsaturated FAs	3.95 %
	Cholesterol	31 ppm

**Minerals**	Ash	5.8 %
	Calcium	0.80 %
	Phosphorus	0.50 %
	Phosphorus (non-phytate)	0.25 %
	Potassium	0.80 %
	Magnesium	0.15 %
	Sulfur	0.25 %
	Sodium	0.43 %
	Chloride	0.70 %
	Fluorine	8.4 ppm
	Iron	170 ppm
	Zinc	110 ppm
	Manganese	120 ppm
	Copper	17 ppm
	Cobalt	0.63 ppm
	Iodine	1.45 ppm
	Chromium (added)	0.02 ppm
	Selenium	0.30 ppm
**Vitamins**	Carotene	0.2 ppm
	Vitamin K	3.0 ppm
	Thiamin (B1)	12.5 ppm
	Riboflavin (B2)	5.5 ppm
	Niacin	75 ppm
	Pantothenic Acid	20 ppm
	Choline	1500 ppm
	Folic Acid	2.9 ppm
	Pyridoxine (B6)	9.6 ppm
	Biotin	0.30 ppm
	Vitamin B12	51 mcg/kg
	Vitamin A	18 IU/g
	Vitamin D3 (added)	3.3 IU/g
	Vitamin E	66 IU/kg
	Ascorbic Acid (Vitamin C)	0.00 mg/g
**Caloric Distribution**	From Protein	19.896 %
	From Fat (ether extract)	26.088 %
	From Carbohydrates	54.016 %

[1]Formulation based on calculated values from the latest ingredient analysis information. Since nutrient composition of natural ingredients varies and some nutrient loss will occur due to manufacturing processes, analysis will differ accordingly.
[2]Nutrients expressed as percent of ration except where otherwise indicated. Moisture content is assumed to be 10.0% for the purpose of calculations.
[3]NDF = approximately cellulose, hemi-cellulose and lignin.
[4]ADF = approximately cellulose and lignin.
[5]Physiological Fuel Value (kcal/ gm) = Sum of decimal fractions of protein, fat and carbohydrate (use Nitrogen Free Extract) x 4,9,4 kcal/ gm respectively.

Table S2. Composition of the Western Diet (Teklad: TD.88137) used in experimental groups.

**Formula (g/kg)**		
	Nutrient/Component	*Amount*
	Casein	195.0
	DL-Methionine	3.0
	Sucrose	341.46
	Corn Starch	150.0

	Andydrous Milkfat	210.0
	Cholesterol	1.5
	Cellulose	50.0
	Mineral Mix, AIN-76 (170915)	35.0
	Calcium Carbonate	4.0
	Vitamin Mix, Teklad (40060)	10.0
	Ethoxyquin	0.04
**Selected nutrient information[1]**		
	% by weight	*% kcal from*
Protein	17.3	15.2
Carbohydrate	48.5	42.7
Fat	21.2	42.0
Cholesterol[2]	0.2%	
**Typical fatty acid analysis, % of diet[3]**		
	Mean	*SD*
Total	20.7	1.5
Saturated fat	12.8	0.8
Monounsaturated fat	5.6	0.5
Polyunsaturated fat	1.0	0.2
Unknown[4]	1.3	0.3
**Typical fatty acid profile, % of total fatty acids[3]**		
	Mean	*SD*
Saturated fat	61.8	2.0
Monounsaturated fat	27.3	2.1
Polyunsaturated fat	4.7	0.8
4:0	2.1	1.1
6:0	1.5	0.7
8:0	1.1	0.3
10:0	2.6	0.5
12:0	3.3	0.5
14:0	10.6	0.9
16:0	28.9	1.3
16:1	1.5	0.2
18:0	12.5	0.8
18:1 (Oleic)	20.9	2.6
18:1 Isomers[5]	4.0	1.2
18:2 (Linoleic)	2.3	1.0
18:2 Isomers[6]	1.3	0.5
18:3 (Linolenic)	0.7	0.2

[1] *Values are calculated from ingredient analysis or manufacturer data.*
[2] *0.15% added, 0.05% from fat source.*
[3] *n = 21, analysis conducted by two independent laboratories.*
[4] *Unidentified fatty acids and those contributing on average less than 0.5% of total fatty acids.*
[5] *Includes trans isomers elaidic and vaccenic acid and unidentified cis isomers.*
[6] *Includes trans isomers.*

Table S3. RNA samples and sequencing data quality summary for all samples in the study.

Sample ID	RIN	Raw reads	Raw data	Effective (%)	Error (%)	Q20 (%)	Q30 (%)	GC (%)	Sex	Age at collection time (days)	Diet
F6	7.6	47480236	7.1	95.22	0.03	97.93	94.09	46.44	female	530	Normal
F7	7.2	41040518	6.2	92.62	0.03	97.91	94.10	46.39	female	530	Normal
F8	5.9	40542370	6.1	93.89	0.03	97.84	93.78	46.04	female	530	Normal
F9	7.3	39506202	5.9	94.32	0.03	97.67	93.50	47.30	female	530	Normal
F11	6.4	52640650	7.9	94.48	0.03	97.81	93.87	46.11	female	530	Western
F12	6.9	44380528	6.7	92.94	0.02	97.99	94.22	46.77	female	530	Western
F13	7.5	61397196	9.2	94.12	0.03	97.86	93.98	46.94	female	530	Western
F14	7.2	52730940	7.9	95.71	0.03	97.89	94.01	46.44	female	530	Western
F15	8.1	47582722	7.1	97.30	0.03	97.94	94.12	47.29	female	530	Western
F26	6.0	57791222	8.7	95.77	0.03	97.83	93.93	46.72	female	640	Normal
F27	5.4	57058412	8.6	96.61	0.03	97.90	94.10	46.56	female	640	Normal
F28	4.9	53974628	8.1	93.67	0.03	97.77	93.88	45.85	female	640	Normal
F29	4.1	40212506	6.0	97.47	0.03	97.63	93.61	46.46	female	640	Normal
F30	5.6	61980142	9.3	96.92	0.03	97.82	93.97	46.51	female	640	Normal
F31	7.7	54959646	8.2	95.22	0.03	97.80	93.77	48.34	female	640	Western
F32	6.9	44026534	6.6	97.17	0.03	97.88	94.03	46.08	female	640	Western
F33	6.8	48023452	7.2	96.75	0.03	97.77	93.81	46.25	female	640	Western
F34	6.2	49463808	7.4	96.58	0.03	97.79	93.83	46.46	female	640	Western
F35	6.5	47279372	7.1	96.68	0.03	97.74	93.81	46.03	female	640	Western
M6	5.4	58857082	8.8	95.95	0.02	98.01	94.41	47.28	male	530	Normal
M7	6.7	58064074	8.7	96.92	0.03	97.74	93.94	47.83	male	530	Normal
M8	4.2	56044176	8.4	95.86	0.03	97.66	93.81	46.87	male	530	Normal
M9	4.4	62833448	9.4	97.52	0.03	97.44	93.51	46.88	male	530	Normal
M10	6.0	46683450	7.0	96.93	0.03	97.81	93.97	47.03	male	530	Normal
M11	3.9	59752882	9.0	96.45	0.03	97.53	93.52	47.27	male	530	Western
M12	5.0	53005760	8.0	96.05	0.03	97.89	94.09	46.71	male	530	Western
M13	5.6	51982606	7.8	95.35	0.03	97.78	93.90	46.75	male	530	Western
M14	5.3	41066522	6.2	96.37	0.03	97.74	93.87	46.65	male	530	Western
M15	5.7	48638506	7.3	96.13	0.03	97.74	93.78	46.82	male	530	Western
M26	5.1	49121604	7.4	97.55	0.03	97.3	93.20	45.68	male	750	Normal

M27	7.3	40129056	6.0	95.32	0.03	97.59	93.47	47.44	male	750	Normal
M28	3.6	48900676	7.3	97.86	0.03	97.06	92.92	45.48	male	750	Normal
M29	3.6	49081328	7.4	96.23	0.03	96.76	92.49	45.71	male	750	Normal
M30	5.7	47638898	7.1	96.54	0.03	97.41	93.46	45.19	male	750	Normal
M31	6.6	47038118	7.1	96.60	0.03	97.61	93.60	46.77	male	750	Western
M32	3.8	50924890	7.6	97.40	0.03	97.12	92.94	45.55	male	750	Western
M33	7.2	40032018	6.0	95.45	0.03	97.38	93.08	47.15	male	750	Western
M34	7.5	58372882	8.8	97.45	0.03	97.57	93.57	47.14	male	750	Western
M35	6.3	45787344	6.9	95.12	0.03	97.67	93.79	46.44	male	750	Western

Raw reads: *total amount of reads of raw data each four lines taken as one unit (amount of read1 and read2).*

Raw data: *(Raw reads) * (sequence length:150) calculating in G.*

Effective: *(Clean reads/Raw reads) *100%.*

Error: *base error rate.*

Q20 Q30: *(Base count of Phred value > 20 or 30) / (Total base count).*

GC: *(G & C base count) / (Total base count).*

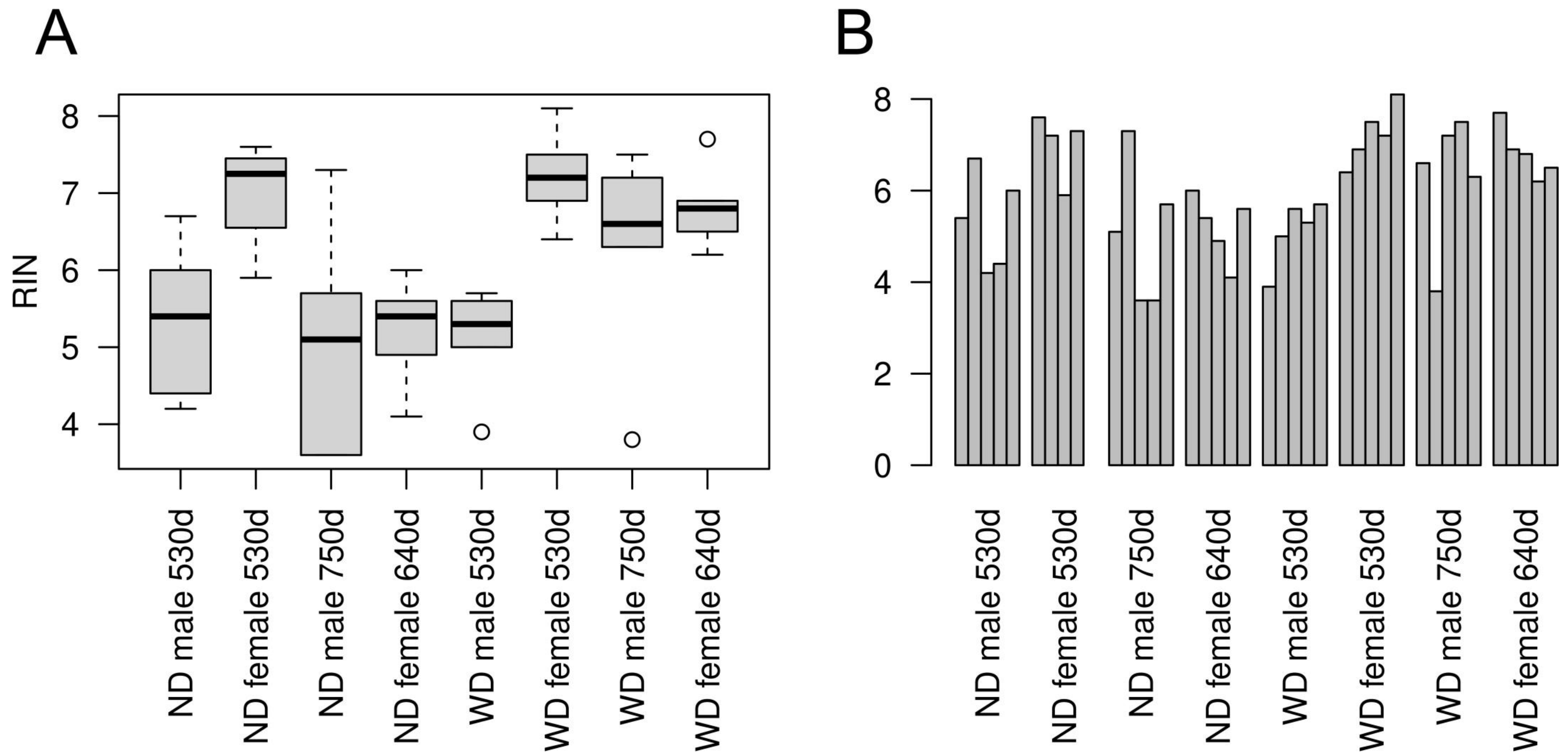

Figure S1. RNA integrity numbers (RIN) of unique experimental groups. The RIN values of RNA samples are presented as box plots (A) and bar plots (B).

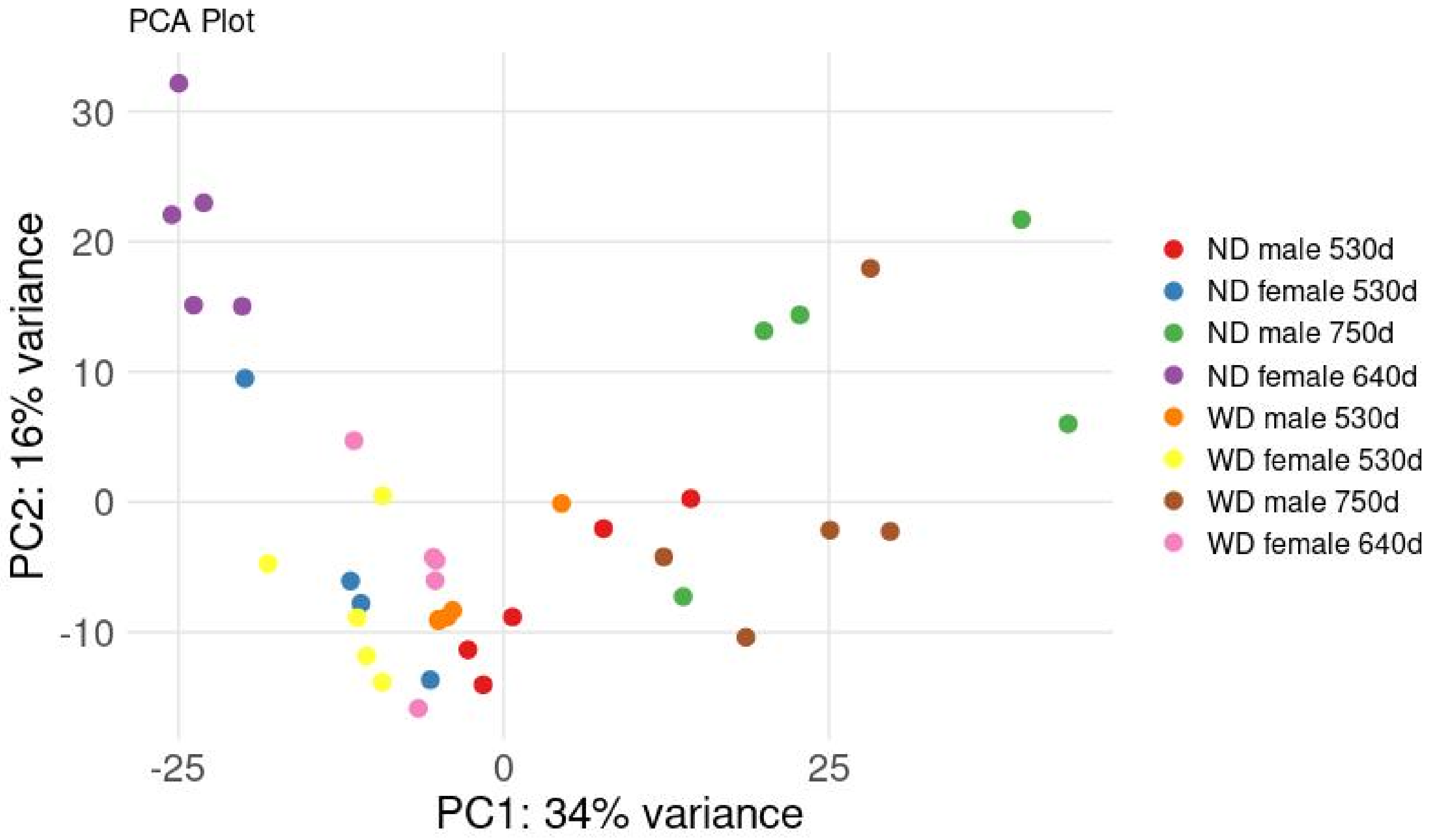

Figure S2. Principal component analysis by diet, sex, and collection age

PCA of transcriptomic profiles from all samples, colored by group: normal diet (ND) male 530d (red), ND female 530d (blue), ND male 750d (green), ND female 640d (purple), western diet (WD) male 530d (orange), WD female 530d (yellow), WD male 750d (brown), and WD female 640d (pink). Each point represents one sample. PC1 and PC2 explain 34% and 16% of the variance, respectively.

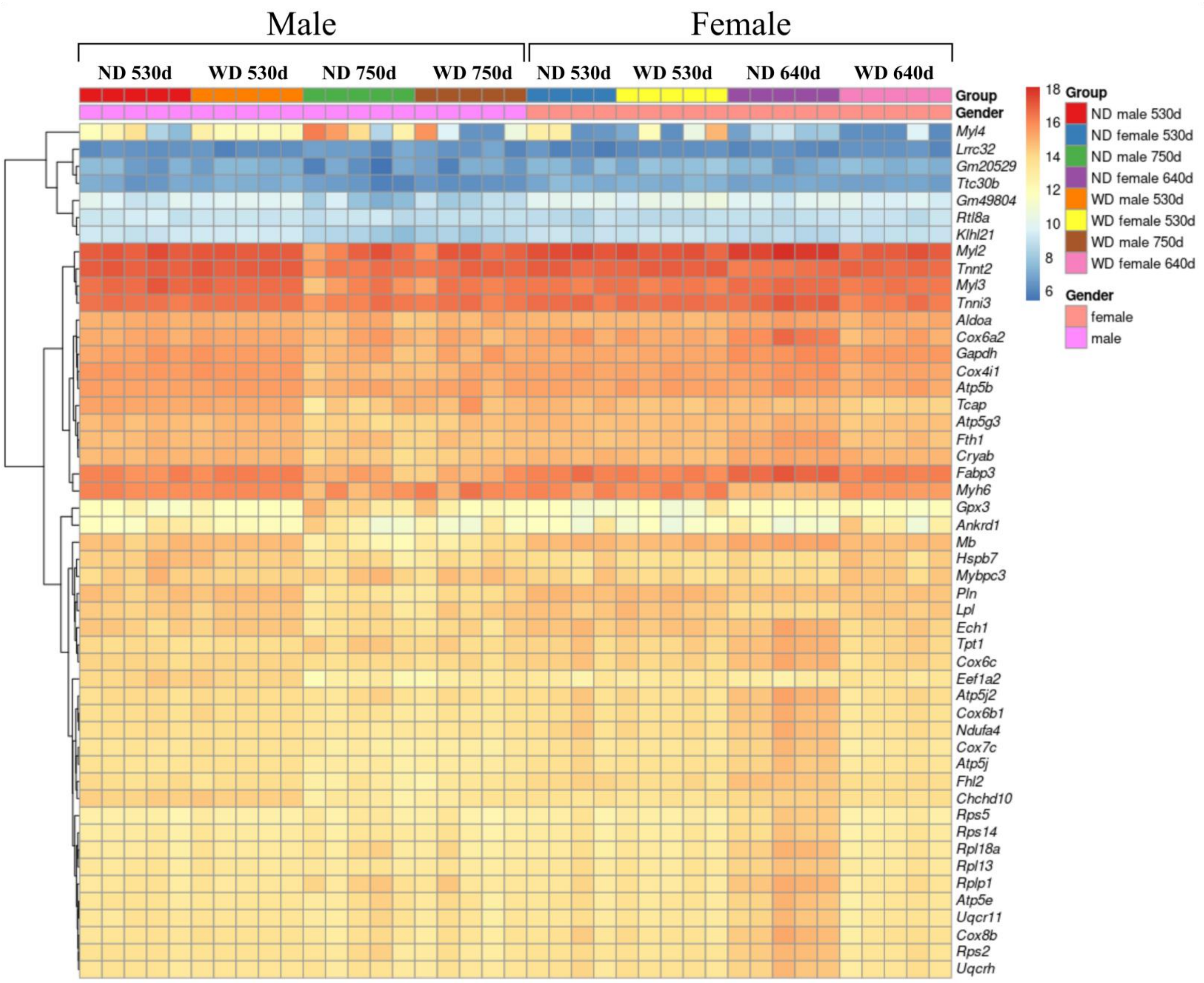

Figure S3. Heatmap of highly variable genes across diet, sex, and collection age

Heatmap of the top 50 highly variable genes across samples, grouped by diet (normal diet (ND) vs western diet (WD)), sex, and collection age (530days, 640days/750days). Columns are annotated by sex and age category.
